# Pediatric surgery in Burkina Faso: capacity, gaps, and challenges

**DOI:** 10.1007/s00383-025-06130-7

**Published:** 2025-07-25

**Authors:** Sophie Inglin, Abou Coulibaly, Yasmine Yousef, Anata Bara, Seni Kouanda, Barbara E. Wildhaber

**Affiliations:** 1 Institute of Global Health, University of Geneva, Geneva, Switzerland; 2 Division of Pediatric and Adolescent Surgery, Department of Pediatrics, Gynecology, and Obstetrics, University Hospitals of Geneva, University of Geneva, Rue Willy Donzé 6, 1205 Geneva, Switzerland; 3 Biomedical and Public Health Department, Research Institute of Health Sciences, National Center for Scientific and Technological Research, Ouagadougou, Burkina Faso; 4 Joe DiMaggio Children’s Hospital, Memorial Healthcare System, Hollywood, FL USA; 5 Ministry of Health, Ouagadougou, Burkina Faso

**Keywords:** Pediatric surgery, Assessment, Challenges, Low-to-middle-income countries

## Abstract

**Purpose:**

Burkina Faso faces challenges in pediatric surgical care, with apparent gaps in human resources, infrastructure and education. However, no quantitative analysis has been performed to date. This study aims to evaluate the capacity for pediatric surgery across Burkinabé healthcare facilities.

**Methods:**

This study is a cross-sectional descriptive analysis conducted in 26 healthcare facilities in Burkina Faso, including first-, second-, and third-level hospitals as well as private clinics. It used the Global Assessment in Pediatric Surgery (GAPS) tool to evaluate five domains: human resources, material resources, outcomes, accessibility, and education.

**Results:**

The study showed significant gaps in pediatric surgical care: A full-time pediatric surgeon is appointed in 7/26 facilities. Specialized infrastructure for pediatric surgery is limited: Operating rooms dedicated to pediatric surgery are observed in 2/6 tertiary hospitals, minimally invasive surgery is available in 3/26 facilities. Education programs were deficient: Continuing medical education to > 50% of professionals is offered in 1/26 facility. 22/26 facilities provided follow-up care for more than 50% of their patients.

**Conclusion:**

Burkina Faso faces major challenges in pediatric surgical care, particularly related to human and material resources as well as training. These issues must be addressed in the upcoming national pediatric surgery strategy to ensure equitable access to quality care across the country.

## Introduction

Sub-Saharan Africa confronts a significant public health challenge, with the region bearing the world’s highest unmet surgical needs, estimated at approximately 41 million cases annually [1]. The Lancet has described this critical situation as a silent killer, highlighting the urgent need to recognize surgery as a fundamental component of a functional and resilient health system [2]. Indeed, surgery alone has the potential to address up to one-third of the global disease burden [3].

According to UNICEF's *Generation 2030 Africa 2.0* report, 47% of Africa's population is under the age of 18, and this number is expected to increase by 170 million by 2030 [4]. These children have an 85% risk of requiring surgical care by the age of 15 years old [5]. Given this context, strengthening access to surgical and anesthesia services for children is essential, not only to enhance health outcomes but also to ensure the survival and successful reintegration of these young individuals into their communities [6].

Several sub-Saharan African countries have developed National Surgical, Obstetric, and Anesthesia Plans (NSOAPs) to systematically address needs in surgical care and improve outcomes, reinforce health systems and promote fair access to essential surgical services as a fundamental element of achieving universal health coverage [7, 8]. Integrated within wider health system development strategies, NSOAPs function as critical policy instruments, helping to steer national investments in surgical care and embed life-saving interventions [8]. While pediatric surgical capacity has been studied in some African settings [9], comprehensive data are lacking for Burkina Faso. This study evaluates pediatric surgical capacity across Burkina Faso’s health facilities to identify gaps and provide essential evidence supporting the development of a national pediatric surgery strategy, aligning with regional efforts to enhance access to safe and timely surgical care for children.

## Methods

This study is a cross-sectional, descriptive study conducted to evaluate pediatric surgical capacities in Burkina Faso using the Global Assessment in Pediatric Surgery (GAPS) tool [10, 11].

### The GAPS tool

The GAPS tool, developed by Yousef et al. in 2019, has been tested in 65 institutions of eight countries (Somaliland, Democratic Republic of Congo, Uganda, Mongolia, Egypt, Vietnam, Algeria, Thailand) and showed to be valuable for identifying resource gaps, shaping policy, and improving pediatric surgical care, particularly in low-resource settings [10]. Version 3 of the tool includes a short version with 64 items that assesses pediatric surgical systems across five dimensions: human resources (9 variables), material resources (39 variables), outcomes (3 variables), accessibility (3 variables), and education (10 variables). In this study, we used the long version of the tool, which includes 168 items, to gather in-depth insights into pediatric surgical capacities.

### Data collection

The study included all hospitals in Burkina Faso that provide pediatric surgical care and have general anesthesia capabilities. We thus assessed all tertiary-level university hospitals, all regional hospital centers, some private clinics meeting the specified inclusion criteria, as well as all primary-level healthcare facilities equipped with surgical units located in Ouagadougou and Bobo-Dioulasso. Data collection was conducted between May 8 and June 10, 2023. Two pediatric surgeons were trained in the data collection techniques by the scientific team at the Institute of Research in Health Sciences in Ouagadougou. The survey was carried out through (i) face-to-face interviews with various hospital staff members, including hospital directors, (ii) extraction of information in health facilities records, and (iii) observation of map-related questions. Additionally, the hospital visits involved guided tours of the facilities, including operating rooms and wards. Authorization for each visit was approved by the hospital director. No individual patient data was collected from the visited healthcare facilities. The Burkina Faso’s National Ethics Committee of Health Research approved the study (N°2023-03-049).

### Data analysis

Descriptive statistical analyses were computed using Stata 18.0 (StataCorp, College Station, TX). We presented numbers and proportions for each of the items of GAPS. GAPS Score and radar plots were calculated and generated as previously described by Yousef et al. [11].

## Results

### Overall results

Twenty-six facilities were included in the study. Data from 4 first-level hospitals, 9 secondary-level hospitals, 6 tertiary-level hospitals, and 7 private clinics were collected exhaustively.

#### Human resources

Table 1 summarizes the results of the GAPS-domain human resources. Among the hospitals, 11/26 facilities reported having a pediatric surgeon on staff during the past month, including four private clinics. However, only 7/26 facilities reported having a full-time pediatric surgeon, the majority of whom are based in tertiary-level healthcare facilities. Trained anesthesiologists with experience in pediatric anesthesia but no formal subspecialty in pediatric anesthesiology training are available in 21 facilities. The analysis also underscores the near-total absence of pediatric intensivists and pediatric emergency physicians across all healthcare facilities in the country. Furthermore, only one hospital—a secondary-level healthcare facility—reported having a pediatric radiologist. Lastly, no healthcare facility had over 50% of its nursing staff trained in pediatrics, indicating that most children receive care from nurses who lack specialized pediatric training.

**Table 1:** Results of the GAPS-domain *human resources* by level of healthcare facility

Human resources	First-level Hospital (4)	Second-level Hospital (9)	Third-Level Hospital (6)	Private Clinic (7)	Total (26)
Presence of children's surgeon (trained surgeon with formal specialized training in children’s surgery of > 1 year) (yes)	0	2	5	4	11
Presence of pediatric general surgeon/urologist full time (trained surgeon with formal specialized training in pediatric surgery of > 1 year) (yes)	0	1	4	2	7
Presence of pediatric orthopedic surgeon full time (yes)	0	0	1	0	1
Presence of pediatric otolaryngologist/maxillofacial surgeon full time (yes)	0	0	0	0	0
Presence of pediatric cardiac surgeon full time (yes)	0	0	0	0	0
Presence of pediatrician full time (Medical doctor with formal specialization of > 1 year in pediatrics/neonatology) (yes)	3	7	6	2	18
Trained anesthesiologist with experience in pediatric anesthesia but no formal subspecialty pediatric anesthesiology training (yes)	1	8	6	6	21
Presence of pediatrician working in Pediatric Intensive Care Unit/Neonatal Intensive Care Unit with no formal specialization in pediatric/ neonatology intensive care (yes)	1	6	3	2	12
Trained emergency physician with no formal pediatric expertise (yes)	0	4	1	0	5
Pediatric radiologist on-site (yes)	0	1	0	0	1
Pediatric pathologist on-site (yes)	0	0	0	0	0
> 50% of the nurses caring are pediatric nurses	0	0	0	0	0
> 50% of the nurses caring for children hold a bachelor's degree in nursing	4	9	6	7	26

Reported answers are in parenthesis after the question

#### Infrastructure and material resources

Table 2 summarizes results of the GAPS-domain material resources. While most hospitals reported having access to running water during the previous month (20/26), only one-third of tertiary hospitals reported such access. In contrast, most facilities (24/26) have access to public electricity (Table 2). Only 1 out of 26 facilities in the country is exclusively dedicated to pediatric care. While most healthcare facilities (20/26) have at least one operating room available for pediatric surgeries, only 2/6 tertiary hospitals declared to have operating rooms specifically dedicated to pediatric surgery, a minimum of ten beds allocated for pediatric surgery and a recovery unit exclusively for children.

**Table 2:** Results of the GAPS-domain *material resources* by level of healthcare facility

Material resources	Primary-Level Hospital (4)	Secondary-Level Hospital (9)	Third-Level Hospital (6)	Private Clinic (7)	Total (26)
Is this a dedicated children's hospital? (yes)	0	0	1	0	1
Capacity to do FAST ultrasounds/X-rays in the emergency department? (yes)	0	6	2	4	12
Is there a maternal unit attached or associated to your institution? (yes)	4	9	5	4	22
Does your institution have capacity to perform cesarean sections? (yes)	4	9	5	7	25
Hospital beds dedicated to children's surgery? (> 10)	0	0	2	0	2
Operating rooms *dedicated* to children's surgery? (1 to 2)	0	0	2	0	2
Operating rooms *available* to children's surgery? (At least 1)	3	8	3	6	20
Mechanical ventilation is provided in the operating room? (yes)	3	9	6	6	24
Area dedicated to a post-operative recovery unit available (yes)	3	9	6	7	25
Area in the post-operative recovery unit dedicated to children only (yes)	0	0	2	1	3
Area dedicated to critically ill patients (yes)	0	1	1	0	2
ICU/PICU/NICU beds are available to pediatric/neonatal patients? (> 4)	0	1	0	0	1
External (or public) electricity (yes)	4	8	5	7	24
Functioning CT scan (yes)	0	4	4	3	11
Functioning anesthesia machine (yes)	3	9	6	7	25
Functional neonatal incubators the institution have access (at least 4)	0	2	1	1	4
Functional ventilators for neonates the institution have access (at least 4)	0	0	1	0	1
Functional ventilators for children the institution have access (at least 4)	0	0	2	0	2
Supplemental oxygen available to children undergoing general anesthesia? (yes)	4	7	4	6	21
Defibrillator available in each operating room (yes)	0	0	0	1	1
Electrocautery (yes)	4	9	6	6	25
Functioning Laryngoscope Handle and Pediatric sized blades (yes)	3	7	3	5	18
Pediatric endotracheal tubes (2.5-6 mm) (yes)	0	7	2	5	14
Pediatric oral airways (000–4) (yes)	0	7	2	5	14
Pediatric nasal airways (yes)	0	8	2	5	15
Sterilized surgical instrument sets for open surgery (yes)	4	6	3	5	18
Sterilized surgical instrument sets for emergency thoracotomy (yes)	0	0	2	3	5
Functioning autoclave (sterilizer) (yes)	4	9	5	7	25
Capnograph (yes)	4	6	3	4	17
Electric warming blanket (yes)	4	9	6	7	26
Depth of anesthesia monitor (i.e. BIS) (yes)	0	0	0	1	1
Pediatric chest tubes (< 20 Fr) (yes)	0	1	1	3	5
Functioning chest drainage system (yes)	0	0	0	2	2
Anticonvulsants (yes)	0	1	2	3	6
Hypertonic saline (3%) (yes)	3	5	5	6	19
Parenteral Nutrition (i.e. amino-acids, Intralipid) (yes)	0	5	0	2	7
Paralytics (i.e. Rocuronium, succinylcholine, and more.) (yes)	1	7	3	6	17
Vasopressors (i.e. norepinephrine, epinephrine, dopamine, and more) (yes)	4	8	3	6	21
On-site blood bank (yes)	1	6	6	2	15
Availability of running water (yes)	3	8	2	7	20
Availability of laboratory services (yes)	4	9	6	7	26
Functional and available X-ray machine (yes)	1	8	6	6	21
Functional and available ultrasound machine (yes)	0	8	6	5	19
Resuscitation equipment available last month (yes)	2	7	3	5	17
Sutures 4–0 or 0.15 mm available last month (yes)	1	7	4	6	18
Pediatric urinary catheter (< 10 Fr) available last month (yes)	1	3	3	4	11
Pediatric central venous catheters available last month (yes)	0	0	0	2	2
Broad-spectrum antibiotics (yes)	4	8	2	6	20

Reported answers are in parenthesis after the question

More than half of secondary-level hospitals (7/9) and private clinics (5/7) are equipped with critical pediatric surgical tools, including functioning laryngoscope handles, endotracheal tubes, or oral airways. These types of facilities are also proportionally better equipped with sterilized surgical instrument sets for open surgery, paralytics, vasopressors, and broad-spectrum antibiotics.

#### Outcomes

Table 3 summarizes the results of the GAPS-domain outcomes. Almost all health facilities (22/26) report a follow-up rate after major procedures exceeding 50%. In terms of monitoring, 22/26 facilities continuously track airway and ventilation. However, only 12/26 facilities use capnography in more than 50% of cases to assess ventilation—this includes only one-third of tertiary-level hospitals (2/6) compared with two-thirds (6/9) of secondary-level hospitals.

**Table 3:** Results of the GAPS-domain *outcomes* by level of healthcare facility

Outcomes	Primary-Level Hospital (4)	Secondary-Level Hospital (9)	Third-Level Hospital (6)	Private Clinic (7)	Total (26)
The 30-day follow-up rate after major procedures (> 50%)	1	9	6	6	22
The airway and ventilation continuously monitored (> 50%)	1	9	6	6	22
Adequacy of ventilation monitored with capnography (> 50%)	1	6	2	3	12

Reported answers are in parenthesis after the question

#### Accessibility

Table 4 summarizes the results of the GAPS-domain accessibility. Very few (3/26) facilities are equipped to perform minimally invasive surgeries; of those, 2 are private clinics, and 1 is a tertiary-level hospital. Additionally, 4/6 tertiary-level hospitals are capable of operating on pediatric patients classified as ASA class 3 and above, and none of the other healthcare facilities are capable of doing so.

**Table 4:** Results of the GAPS-domain *accessibility* by level of healthcare facility

Accessibility	Primary-Level Hospital (4)	Secondary-Level Hospital (9)	Third-Level Hospital (6)	Private Clinic (7)	Total (26)
People the institution serves (in millions)					
• < 1 million	4	4	0	3	11
•1–10 millions	0	5	2	3	10
•10–20 millions	0	0	3	1	4
• > 20 millions	0	0	1	0	1
Does your institution perform minimally invasive surgery? (yes)	0	0	1	2	3
Highest ASA class of children operated at the institution (= 3 and above)	0	0	4	0	4

Reported answers are in parenthesis after the question.

#### Education

Table 5 summarizes the results of the GAPS-domain education. Only 7/26 hospitals are affiliated with a university or medical school, comprising the totality of third-level hospitals and 1 secondary-level hospital. In terms of training, 4/6 third-level hospitals involve surgical residents in pediatric care and offer formal training in children's surgery and anesthesiology. However, only in 1/26 facilities (a second-level hospital) do more than 50% of professionals participate in continuing medical education annually. Furthermore, ongoing research is limited to just 1 tertiary hospital, and only 2 third-level hospitals (2/6) have a dedicated clinical unit for children after surgery.

**Table 5:** Results of the GAPS-domain *education* by level of healthcare facility

Education	Primary-Level Hospital (4)	Secondary-Level Hospital (9)	Third-Level Hospital (6)	Private Clinic (7)	Total (26)
Is your institution affiliated with a university or medical school (yes)	0	1	6	0	7
Surgical residents/registrars involved in the care of pediatric patients(yes)	0	0	4	0	4
Are surgeons undergoing formal training in children's surgery involved (yes)	0	0	4	0	4
Institution has a formal surgical residency/registrar training program (yes)	0	0	4	0	4
Institution has any formal fellowship in children's surgical specialties (yes)	0	0	3	0	3
Institution has a formal anesthesiology training program (yes)	0	0	5	0	5
≤ 50% of professionals involved attend continuing medical education events/year (yes)	0	1	0	0	1
Ongoing research projects does the department of surgery have (= 5 and above)	0	0	1	0	1
Oncological cases regularly discussed at an interdisciplinary tumor board (yes)	0	1	0	0	1
Is there a clinical unit dedicated solely to children's surgery? (yes)	0	0	2	0	2

Reported answers are in parenthesis after the question

### Results according to the level of healthcare facility

**Fig. 1:**
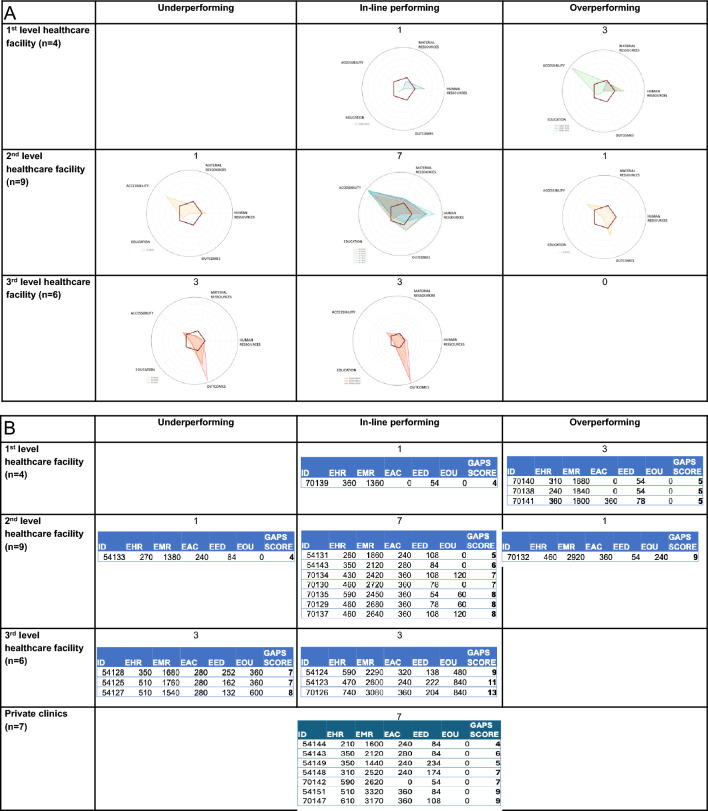
**A** Radar plots: performance of healthcare facilities according to the GAPS’s domains. The wider the spider, the better the performance. **B** GAPS scoring results stratified by care level according to each domain. The higher the score, the better the performance. EHR, human resources; EMR, material resources; EAC, accessibility; EED, education; EOU, outcomes

Table 6 shows an overview of under-, over- and in-line performing healthcare facilities, according to the GAPS scoring system. 3/4 first-level hospitals overperform, due to their better scoring in the domain of material resources (detailed are available in supplementary data Fig. 1a, b). The majority (7/9) of second-level healthcare facilities are in line and meet performance expectations. Half (3/6) of the third-level hospitals performed as expected, while the other half underperformed due to significant deficiencies, particularly in the areas of human resources and equipment. GAPS radar plots, showing the performance of healthcare facilities according to the different domains (Fig. 1A) and GAPS scoring results stratified by care level according to each domain (Fig. 1B) are presented below.

**Table 6:** Overview of *under-, over- and in-line performing* healthcare facilities, according to the GAPS scoring system

	GAPS score by level of hospitals	Total	Underperforming	In-line performing	Overperforming
1st level healthcare facility	≤ 4	4	0	1	3
2nd level healthcare facility	5–8	9	1	7	1
3rd level healthcare facility	9–14	6	3	3	0

## Discussion

Over the past decade, increasing awareness of inequitable access to pediatric surgical services has driven global research and advocacy efforts [12]. A pivotal advancement in these efforts has been the development of pediatric surgery assessment tools, which offer a comprehensive overview of the readiness and capabilities of healthcare facilities to deliver pediatric surgical care.

Our study highlights a critical shortage of human resources for pediatric surgery in Burkina Faso, a country with a population of over 9.5 million children. Only 7/26 facilities reported having a full-time pediatric surgeon on staff during the month preceding the assessment, with the majority of these surgeons concentrated in tertiary hospitals located in the capital city. This corresponds to an approximate ratio of 0.07 pediatric surgeons per 100,000 children, which is well below the international recommendation of at least 1 qualified pediatric surgeon per 100,000 children (1/3). This raises significant concerns about equitable access to specialized care, particularly for children in rural or underserved regions, leaving many areas without adequate pediatric surgical expertise. This situation mirrors the broader challenges across sub-Saharan Africa, where there is, on average, only one pediatric surgeon for every six million children aged 0 to 14 [13]. To address this challenge, Burkina Faso, like many other countries in the region, relies on general surgeons to perform pediatric surgery. In Burkina Faso, this mainly concerns second-level hospitals, which also refer all children under five years old to third-level hospitals. However, efforts to address this issue are underway in Burkina Faso; since 2017, an advanced diploma in pediatric surgery has been offered and 40 additional scholarships were approved by the Council of Ministers in 2024. These initiatives represent a promising step toward strengthening the country’s pediatric surgical workforce and improving access to care.

While Burkina Faso has a significant number of anesthesiologists with good experience in the field, some with less than a year of training, none have undergone specialized dedicated training in pediatric anesthesia for over a year. This reflects broader global challenges identified by Kempthorne et al. in 2017, who link higher anesthesia-related mortality in low-resource settings to insufficient specialized training and resources, emphasizing the critical need for further development in this area [14].

Furthermore, less than 50% of nursing staff are specialized in pediatric care, which highlights a significant gap in expertise for delivering high-quality pre- and postoperative care. This issue is concerning as it appears that most nurses learn on the job and are not prepared to care for children. Aware of this challenge, Burkinabé health authorities have supported the implementation of a specialized training program for nurses, training over 350 professionals between 2020 and 2024, notably through the pediatric surgery development plan implemented with the Geneva University Hospitals [15]. Moreover, the basic curriculum for nurses has been supplemented with new teaching units specific to pediatric surgery.

The condition of infrastructure and material resources presents a significant challenge in Burkina Faso, as in many other countries in low resource settings [16], while efforts to expand infrastructure in this field are crucial, as highlighted by Ameh et al. [17].

With only one facility in the entire country being fully dedicated to pediatrics and a mere two facilities equipped with operating rooms and postoperative recovery units dedicated to children in the public sector, the situation underscores the neglect of specialized pediatric infrastructure, which is essential to perform quality and safe surgery [6]. The limited prioritization of pediatric-specific surgical needs, even in tertiary institutions, leads to delays in the management of children needing surgery. Of interest, our study’s findings highlight a notable deficit in material resources at tertiary hospitals, especially compared to secondary-level facilities and private clinics. This disparity, which is most likely often due to the reliance on faith-based or partner-supported secondary institutions, underscores a significant gap in essential equipment at higher-level hospitals, which should ideally be best equipped to manage complex pediatric cases. It should also be added that level-2 regional hospitals received substantial support through a government policy to respond to COVID-19, including a significant amount of equipment, particularly for critical care.

The assessed GAPS outcome parameters of pediatric surgical care in Burkina Faso show a mixed picture. Out of 26 facilities, 22 reported a 30-day follow-up rate above 50% after major procedures. This suggests that the majority of facilities maintain postoperative follow-up for a significant proportion of patients, reflecting efforts to ensure continuity of care. However, the fact that only 46% of institutions utilize capnography for ventilation monitoring raises concerns about the adequacy of respiratory care practices in a significant portion of the healthcare system. While follow-up rates in pediatric surgery are promising in Burkina Faso, the limited use of capnography, recognized as an essential safety tool, raises concerns, as its absence could contribute to higher risks of respiratory complications and poor outcomes in low-resource settings [18]. The limited implementation of minimally invasive techniques further highlights barriers to surgical gold-standard access, as these modalities are only available in one of the six tertiary-level hospital and in two private clinics (Table 4). This lack of advanced surgical capabilities restricts the range of conditions that can be efficiently treated. Similarly, a study on laparoscopic surgery in low- and middle-income countries identifies challenges such as insufficient training and institutional capabilities, which further hinder the widespread adoption of these techniques [19].

Finally, education and training are critical to improving pediatric surgery capabilities. While some third-level hospitals are affiliated with universities and offer formal training programs, our study shows that the overall participation in continuing medical education in Burkina Faso is low. This suggests a need for systemic improvements to ensure that healthcare professionals stay updated on best practices in pediatric care. Indeed, as evidenced in many low- and middle-income countries, the lack of comprehensive pediatric surgery residency or fellowship programs presents significant barriers to accessing high-quality care [20].

Last but not least, the observed limited research activities across the country suggest an opportunity to strengthen the evidence-based improvements in pediatric surgery, which could help to guide future reforms and enhance the quality of care.

Upon comparison of the various levels of the healthcare system, in our study, we observed notable differences. Our findings highlight deficiencies in infrastructure and equipment within tertiary-level facilities, which handle the majority of pediatric cases. These high-level facilities do not appear to be adequately equipped to manage the case volume and complexity, but have the most specialized human resources. In terms of surgical volume, only data from the Charles-de-Gaulle Pediatric Hospital in Ouagadougou are considered reliable: the total number of pediatric surgery cases between 2022 and 2024 amounted to 10′ 209, with 72 recorded deaths. However, in other hospitals, data are not disaggregated, and as such, pediatric surgical data cannot be identified. This currently represents a weakness in the health information system.

In contrast, secondary-level hospitals show to have sufficient equipment, largely due to collaborations and partnerships, but, inversely, lack of trained professionals, such as pediatric surgeons and anesthesiologists, most needed to utilize these resources effectively. These observations will hopefully advise the development of the upcoming National Strategy for Pediatric Surgery 2026–2030 of the Burkinabé Ministry of health, to ensure that decisions and resource allocation are optimized, to address these disparities and strengthen the pediatric surgical ecosystem.

## Limitations of this study

This study has certain limitations. Firstly, the GAPS tool collects data pertaining to a rather short period, i.e., the previous month, which may introduce variability, particularly for volatile factors such as the availability of electricity, supplies, or the presence of part-time pediatric surgeons or visiting foreign surgeons practicing pediatric surgery. Further, the GAPS tool shows its limitations in the sometimes binary character of answers: it would have been interesting to know the exact number of pediatrics surgeons or pediatric anesthesiologists of each clinic, than just having the result of hospitals having them. As such, results should be interpreted with a certain caution. Nevertheless, we are certain that the major tendencies are well reflected by the GAPS tool, which has proven its concept. Another potential limitation of the tool lies in the design of certain answers from which the investigator must choose, which do not always align with the specific realities of some contexts. For instance, in Burkina Faso, many anesthetists have undergone pediatric-specific training lasting less than a year, a category not accounted for in the questionnaire. This said, we decided to strictly adhere to the GAPS tool, without conceptualizing the assessment. Lastly, although data were collected through interviews, as outlined in the methods section, and verified as much as possible on-site by the investigators, it must be said that it remained challenging, despite the best of our efforts, to accurately assess the functionality of the identified equipment in the hospitals.

## Conclusion

This study highlights the major challenges faced by pediatric surgical care in Burkina Faso, as identified through the GAPS tool. Our findings expose significant disparities and deficiencies in both human and material resources across the assessed healthcare facilities. For several years, Burkina Faso has been making substantial efforts to analyze and strengthen its pediatric surgical ecosystem through numerous initiatives, however, significant weaknesses still exist. These findings provide the Burkina Faso Ministry of Health with concrete data to guide the upcoming National Pediatric Surgery Strategy 2026–2030. They shed light on key priorities in training, equipment, and care organization to improve equitable access and quality of care for pediatric surgical services nationwide.

## Data Availability

The data that support the findings of this study are available from the corresponding author, SI, upon reasonable request. No datasets were generated or analysed during the current study.
